# A zebrafish *gephyrinb* mutant distinguishes synaptic and enzymatic functions of Gephyrin

**DOI:** 10.1186/s13064-024-00191-5

**Published:** 2024-07-27

**Authors:** Emma J. Brennan, Kelly R. Monk, Jiaxing Li

**Affiliations:** Vollum Institute, Oregon Health & Science University, Portland, OR USA

**Keywords:** Zebrafish, Gephyrin, Glycine receptor, Synapse, Molybdenum cofactor (MoCo), Rheotaxis

## Abstract

Gephyrin is thought to play a critical role in clustering glycine receptors at synapses within the central nervous system (CNS). The main in vivo evidence for this comes from *Gephyrin* (*Gphn*)-null mice, where glycine receptors are depleted from synaptic regions. However, these mice die at birth, possibly due to impaired molybdenum cofactor (MoCo) synthesis, an essential role Gephyrin assumes throughout an animal. This complicates the interpretation of synaptic phenotypes in *Gphn*-null mice and raises the question whether the synaptic and enzymatic functions of Gephyrin can be investigated separately. Here, we generated a *gephyrinb* zebrafish mutant, *vo84*, that almost entirely lacks Gephyrin staining in the spinal cord. *gephyrinb*^*vo84*^ mutants exhibit normal gross morphology at both larval and adult stages. In contrast to *Gphn*-null mice, *gephyrinb*^*vo84*^ mutants exhibit normal motor activity and MoCo-dependent enzyme activity. Instead, *gephyrinb*^*vo84*^ mutants display impaired rheotaxis and increased mortality in late development. To investigate what may mediate these defects in *gephyrinb*^*vo84*^ mutants, we examined the cell density of neurons and myelin in the spinal cord and found no obvious changes. Surprisingly, in *gephyrinb*^*vo84*^ mutants, glycine receptors are still present in the synaptic regions. However, their abundance is reduced, potentially contributing to the observed defects. These findings challenge the notion that Gephyrin is absolutely required to cluster glycine receptors at synapses and reveals a new role of Gephyrin in regulating glycine receptor abundance and rheotaxis. They also establish a powerful new model for studying the mechanisms underlying synaptic, rather than enzymatic, functions of Gephyrin.

**Supplementary Information:**

The online version contains supplementary material available at 10.1186/s13064-024-00191-5.

## Background

Gephyrin is a multifunctional 93-kDa protein with distinct roles in various tissues [1]. In the central nervous system (CNS), Gephyrin plays a crucial role in inhibitory synaptic transmission [2]. Meanwhile, Gephyrin is also essential for molybdenum cofactor (MoCo) synthesis [3], acting downstream of MoCo synthesis 1 (MoCS1) and MoCS2. MoCo-dependent enzymes, such as sulfite oxidase and xanthine oxidase, are critical for proper cellular metabolism. MoCo deficiency in humans leads to death in early infancy and severe neurological defects including seizures, feeding difficulties, cerebral atrophy, and white matter lesions [3, 4]. *Mocs1* and *Mocs2* mice die within the first postnatal week and exhibit smaller body size and neuronal apoptosis [5–7]. Similarly, *Gephyrin (Gphn)*-null mice die perinatally and exhibit a range of neurological defects. *Gphn*-null mice lack glycine receptors (GlyRs) at synapses [8, 9], supporting a synaptic role of Gephyrin to cluster GlyRs. However, given the severe CNS defects caused by MoCo deficiency, it has been challenging to fully separate the synaptic role of Gephyrin from its MoCo enzymatic role.

An intrabody-based approach targeting Gephyrin for degradation in zebrafish led to a 64% reduction of Gephyrin levels in a sparse population of motoneurons [10]. However, GlyRs in these fish were not examined, and it is unclear whether a 64% reduction is sufficient to abolish Gephyrin’s function. In vitro assays have been more successful in dissecting the synaptic function of Gephyrin, as disruption of Gephyrin in cultured neurons leads to abolished GlyR clustering [9, 11, 12]. Nonetheless, the in vivo relevance of these defects remains unclear. Hence, there is a need for an in vivo approach that can effectively abolish Gephyrin in the CNS without severely affecting other tissues, thereby facilitating a deeper understanding of its role in synapse organization within the CNS.

In zebrafish, *gephyrin* is duplicated such that *gephyrina* (*gphna*) and *gephyrinb* (*gphnb*) orthologs are encoded in the genome. mRNA expression analysis via in situ hybridization indicates that *gphnb* is specific to the CNS, while *gphna* is more widely expressed [13, 14]. Previous work in zebrafish using morpholinos found that disrupting both *gphna* and *gphnb* is necessary to abolish Gephyrin levels in the spinal cord as assessed by antibody staining. This disruption abolished GlyR clustering and led to abnormal coiling behaviors [13]. However, the known off-target effects of morpholinos [15] and the incomplete gene disruption through transient morpholino injection raise questions about whether stable mutants would recapitulate these phenotypes.

Here, we generated a new *gphnb* mutant in zebrafish (*gphnb*^*vo84*^) with nearly absent Gephyrin staining in the spinal cord. Despite increased mortality in late development, many *gphnb*^*vo84*^ mutants developed normally and survived to adulthood. They also exhibited normal activity of xanthine oxidase. Therefore, these mutants establish an excellent in vivo model where the synaptic function of Gephyrin in the CNS can be studied independently of its enzymatic function. These otherwise normal-appearing mutants displayed impaired rheotaxis (the ability to swim against currents). To our surprise, GlyRs, although at reduced levels, still localized to synaptic regions. These results suggest new roles of Gephyrin and refine our understanding of its synaptic clustering function.

## Methods

### Experimental model and subject details

#### Animal care and use

We used the following zebrafish lines: AB, nacre, and *Tg(mbp:GFP-CAAX)* [16]. We generated *gphnb*^*vo84*^ mutants and transgenic *Tg(olig1:mScarlet)* for these studies. The genotypes of fish and the number of sections used for each experiment are indicated in the figure legends. Animals in the fish facility were maintained at 28° C with a 14 h/10 h light/dark cycle. Larvae and juveniles were nurtured with rotifer suspension and dry food (Gemma 75 and 150, respectively). Adults were fed with a combination of brine shrimp and dry food (Gemma 300). Male and female fish at 3 to 12 months-old were mated; larval fish at 3 dpf to 5 dpf were used for experiments.

#### Generation of *gphnb*^*vo84/vo84*^ mutants

**Fig. 1:**
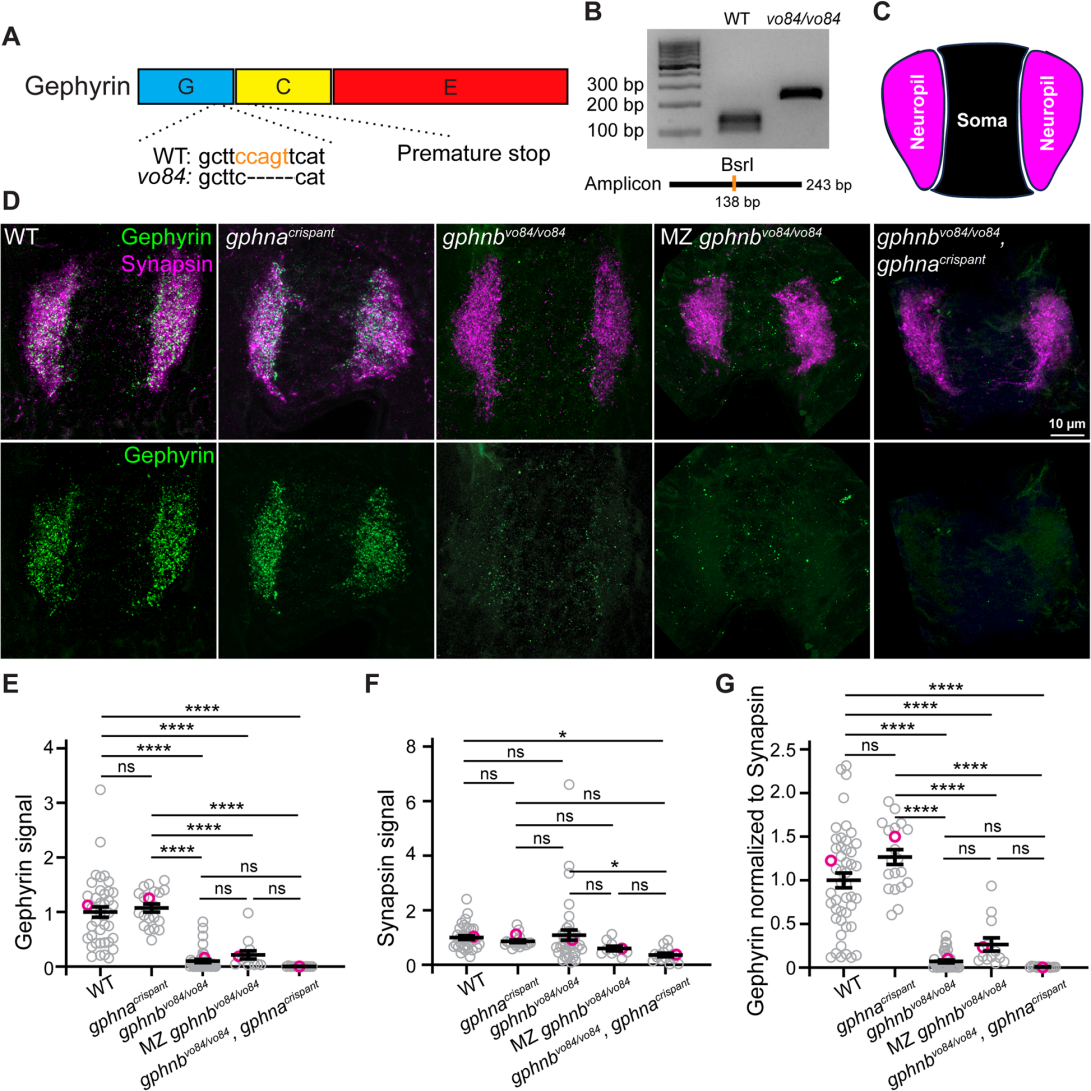
Generation and validation of *gphnb*^*vo84*^ mutants. **A** Schematic of Gephyrin protein showing its three domains and the 5 bp deletion in *gphnb*^*vo84*^ causing a frameshift and premature stop. The BsrI recognition site used for genotyping was indicated in orange. **B** Representative gel images for genotyping *gphnb*^*vo84*^. A BsrI recognition site is disrupted by *gphnb*^*vo84*^ mutation in the middle of the amplicon. **C** Schematic of neuropil (magenta) and soma (black) regions in transverse spinal cord section of zebrafish at 5 dpf. **D** Representative transverse sections in the spinal cord at 5 dpf with Gephyrin (green) and Synapsin (magenta) staining from the following conditions: wildtype (WT); *gphna*^*crispant*^, *gphnb*^*vo84/vo84*^; maternal zygotic (MZ) *gphnb*^*vo84/vo84*^; and *gphnb*^*vo84/vo84*^, *gphna*^*crispant*^. **E**–**G** Gephyrin signal (E), Synapsin signal (F) and Gephyrin normalized to Synapsin (G) from the spinal sections. n_WT_ = 19 fish, 41 sections, 3 technical replicates; n_*gphna*_ = 12 fish, 20 sections, 1 technical replicate; n_*gphnb*_ = 18 fish, 42 sections, 3 technical replicates; n_MZ *gphnb*_ = 7 fish, 13 sections, 1 technical replicate; and n = 8 fish, 15 sections, 1 technical replicate. E, F[4, 126] = 45.29; F, F[4, 116] = 3.303; G, F[4, 129] = 56.29. All data are represented as mean ± SEM; ns, not significant; *, *p* < 0.05; ****, *p* < 0.0001; the data points corresponding to the representative images are noted in pink; ns, not significant; (E–G) one-way ANOVA with Tukey’s post-hoc test; scale bars 10 μm

The sgRNA sequence for *gphnb* exon 7 (GGAGAGCCGGCAGGATGAACTGG) was previously selected and exhibited high cutting efficiency [17]. We injected the sgRNA together with 1 ng Cas9 protein. We sequenced the F1 offsprings from fin clips and found 11 different *gphnb* alleles. We chose the *vo84* allele (*cagtt* deletion within the sgRNA targeting loci) because it causes a frameshift and premature stop codon in an early exon. For genotyping, we used amplicon amplification near the target locus and enzyme digestion. Briefly, the following primers were used to amplify the region near the target locus: Forward TGTCCAGCCCCCTCTGAAACATCC; Reverse TGGAGGGGACGGCAGGTCTTC. The amplicon (243 bp) from wildtype is recognized by BsrI and digested into 106 bp and 137 bp, while the amplicon from *gphnb*^*vo84*^ is not recognized by BsrI (Fig. 1B). These bands can be readily distinguished on 3% gels (Fig. 1B). *gphnb*^*vo84/vo84*^ mutant animals are from *gphnb*^*vo84/*+^ in-crosses; WT in this case refers to the siblings. MZ *gphnb*^*vo84/vo84*^ mutant animals are from *gphnb*^*vo84/vo84*^ in-crosses; WT in this case refers to AB strain.

#### Disruption of *gphna*

The sgRNA sequences for *gphna* exon 2 (GGTCATGAACGAGATCTTTGAGG) and exon 4 (GTGGGACGGGTTTTGCGCCTCGG) were previously selected and exhibited high cutting efficiency [17]. We injected WT or *gphnb*^*vo84/vo84*^ embryos with *gphna* sgRNA and Cas9 protein at the single-cell stage to disrupt *gphna*.

#### Generation of *Tg(olig1:mScarlet)* animals

p5E_olig1 was created using the olig1:Kalta4 plasmid [18], pME_mScarlet was generated using the pME_myrmScarlet plasmid [17], and p3E_polyA was from the Tol2 kit [19]. We injected one-cell stage embryos with 1 nl of an injection solution containing 20 ng µl^−1^ plasmid DNA, 25 ng µl^−1^*Tol2 transposase* mRNA, 0.02% phenol red and 0.2 M KCl. F1 progenies were screened for germline transmission by fluorescence.

#### Tissue sectioning and immunofluorescence

Zebrafish larvae were anesthetized with 0.16 mg/mL tricaine at 5 dpf and then fixed in 4% PFA-0.1% Triton at 4°C overnight with gentle rocking. Heads and posterior tail tips were first removed for better penetration of fixative. The tissue was then washed in PBS-0.1% Triton, passed through 10%, 20%, and 30% sucrose in PBS, and embedded in OCT and frozen at 20 °C. The OCT blocks were cryosectioned into 20 µm transverse slices on Leica Surgipath X-tra slides (3800200). Slides for immunostaining were brought to room temperature for 45 min and rehydrated in PBS-0.1% Triton for one hour. 3% BSA, 5% NGS, 0.2% Triton, and 0.02% sodium azide in 1X PBS was used as blocking buffer. The following primary antibodies were used: mouse Gephyrin mAb7a IgG1 (Synaptic Systems, 1:1000) [17], rabbit Synapsin1/2 (Synaptic Systems, 1:1000) [20], DAPI (Invitrogen, 1:10,000), mouse HuC mIgG2b (Invitrogen, 1:1000) [21], rabbit GlyR IgG1 (mAb4a, Synaptic Systems, 1:500) [22, 23], mouse SV2 mIgG1 (DSHB, 1:1000) [24]. The following secondary antibodies were used at 1:1000: goat anti-rabbit IgG (H + L) Alexa Fluor 488, goat anti-rabbit IgG (H + L) Alexa Fluor Plus 594, goat anti-mouse IgG Alexa Fluor 647. Slides used for GlyR staining were additionally fixed with 95:5 methanol:acetic acid for 10 min at -20°C prior to rehydration. Stained samples were treated with ProLong Glass (Invitrogen P36980) at least one day prior to imaging.

#### Mounting larvae for in vivo imaging

For live imaging experiments, larvae at indicated stages were anesthetized with 0.16 mg/mL (600 µM) tricaine in embryo medium. The fish were mounted in 0.8% low-melt agarose (Sigma A9414) on a coverslip.

#### Confocal microscopy

All fluorescent imaging was performed on an upright Zeiss LSM 980 confocal with Airyscan 2 in 4Y fast mode. For fixed tissues, a 63x/1.4 NA oil objective was used, and for in vivo imaging, a 20x/1.0 NA water objective was used. All images were taken in Zeiss software. The following lasers were used: 488 nm for Alexa488 and GFP, 561 nm for Alexa594, 405 nm for DAPI, and 639 nm for Alexa647. For fixed tissue, z spacing was set to 0.13 µm. For in vivo imaging, z spacing was set to 1.0 µm.

#### Xanthine oxidase activity

Larvae were anesthetized with 0.16 mg/mL tricaine in embryo medium and their tails were collected individually for genotyping. The rest of larval bodies were collected individually in tubes, flash frozen, and stored at -80°C. Subsequently, 15 larvae of the same genotype were pooled together and homogenized with a handheld tissue homogenizer (VWR, 47747–370). The assessment of xanthine oxidase activity was conducted using a commercial kit (Sigma, MAK078-1KT). Fluorescence readings were obtained using a plate reader (CLARIOstarPlus, BMG LABTECH).

#### Swimming behavior assays

##### Coiling behavior recording

At 1 dpf, individual embryos from a *gphn*^*vo84/*+^ in-cross were placed under the scope and recorded in brightfield for 1 min at 40 Hz. The whole embryos were then used for genotyping.

##### Head touch response

At 5 dpf, fish were anesthetized using 0.16 mg/mL (600 µM) tricaine. Subsequently, the fish were transferred to individual wells within a 12-well plate filled with fresh embryo medium. Plates were placed on a hotplate set to 28°C, and the fish were allowed to recover from the effects of the tricaine. Each fish was gently prodded on the head using a probe.

##### Forced group swimming test

Dishes containing 25 mL of embryo medium and 50 5-dpf larvae were placed on a dark background on a 28°C hotplate and allowed to acclimatize. Subsequently, each dish was manually swirled ten times and then recorded for one minute using a Samsung Galaxy S20 phone. The first still was taken after the 11th swirl of embryo medium in the dish, allowing time for the hand to move out of frame after the ten swirls. Subsequent stills were taken every two seconds for eight total timepoints. The Cell Counter plugin in ImageJ was then used to mark the positions of the heads of all fish in each still, as well as the center of the dish. Matlab was used to measure the distance of each fish from the center and quantify the number within each concentric regions of increasing distance from the center at each timepoint.

##### Forced individual swimming test

Dishes containing 25 mL of embryo medium and one 5 dpf larvae were placed in a customized chamber and allowed to acclimatize. The chamber was equipped with 940 nm infrared strip lights and an acA1920-155um camera (Basler 106,879) with a 6 mm C VIS–NIR lens (Edmund Optics 39–939). The images were acquired with pylon Viewer 64-bit (Basler). During the imaging session, each dish was manually swirled three times, kept within a marked area, and recorded for 25 s at a frame rate of 25Hz. The images were processed in ImageJ (1.54f) and the fish was traced with TrackMate (v7.11.1). Fish were subsequently genotyped.

#### Animal survival

Three tanks of 50 fish each of WT and *gphnb*^*vo84/vo84*^ were placed on our system at 5 dpf. Fish were maintained in accordance with standard protocols of our fish facility. Briefly, the tanks are initially half full with no water flow, and the larvae are fed a diet of Gemma 75 and rotifers until 21 dpf. At 21 dpf, water flow is introduced, and fish are switched to Gemma 150 and rotifers. At three months, fish are switched to the adult diet of Gemma 300 and brine shrimp.

### Quantification and statistical analysis

#### Fluorescent signal analysis

After Airyscan processing of confocal images in Zen, all images were analyzed using ImageJ. For fluorescent intensity analyses, maximum projections were generated. For Gephyrin, Synapsin, and GlyR fluorescence, ROIs were drawn delineating the neuropil and soma regions of each projection. The neuropil region was defined by the presence of Synapsin, and the soma region was defined by the lack of Synapsin and presence of DAPI. Integrated density was measured, restricted to the respective ROIs with a standard intensity threshold set for each round of stains. For GFP signal analysis in *Tg(mbp:GFP-CAAX)*, a rectangular ROI of 100-µm length was created around the dorsal region of the spinal cord (body segments 7 or 15). The GFP signal was measured without a threshold.

#### Cell density analysis in the spinal cord sections

We restricted our analysis to a 2 µm section from the center of the z-stack. The Cell Counter plugin in ImageJ was used to mark all cells, identified by nuclear DAPI staining. We manually went through the 2 µm stack to ensure all cells were accounted for. We noted that sometimes HuC fluorescence appeared to colocalize with part of one nucleus but was actually associated with a different nucleus situated above or below. Therefore, HuC + cells were only scored if the HuC fluorescence was visible surrounding the nucleus in all directions to ensure HuC fluorescence is not from any overlapping cells.

#### 3D reconstruction in Imaris

OPCs within a defined 300 µm length of the spinal cord between body segments 7 and 15 in *Tg(olig1:mScarlet)* were analyzed in Imaris. The Surface function was used to identify individual cells (diameter guide 10 µm, surface grain size 0.148 µm, threshold > 700, and quality > 150).

The Spots function was used to identify Gephyrin, GlyR, and SV2 puncta. XY diameter was set to 0.300 µm and Z diameter was set to 0.600 µm. Max intensity threshold was set to at least 1300 for Gephyrin, 2800 for GlyR, and 2200 for SV2. For colocalization analysis, a new set of spots was generated for distance between GlyR and SV2 puncta less than or equal to 0.5 µm, similar to previous work [17]. This set of spots represents colocalization locations between GlyR and SV2. The number of colocalization spots was divided by the total number of GlyR puncta to calculate colocalization ratio.

#### Statistics

All data are shown as mean ± SEM, with individual data points shown as circles. The datapoint that corresponds to the raw data image is indicated in pink. We conducted Shapiro–Wilk’s test and Levene’s test to verify data normality and homoscedasticity, respectively, before performing two-sided *t*-test for unpaired groups, one-way ANOVA test for more than 3 groups, and two-way ANOVA test for more than 1 variable. These analyses were performed in GraphPad Prism. The significance and statistical analyses employed were indicated in figure legends. Animal and cell number used for each experiment, t and df for *t*-tests, and *F[DFn, DFd]* for one-way or two-way ANOVA were indicated in figure legends. In some cases, data collection was not performed blindly due to the experimental conditions, but analyses were performed blindly. Sample size was chosen based on previous studies [17, 25]. Sex is not yet determined in zebrafish at the ages employed in our studies and so cannot be considered as a biological variable.

## Results

### Gephyrin is largely absent in *gphnb*^*vo84/vo84*^ spinal cord

Gephyrin contains three domains: the N-terminal G-domain and the C-terminal C- and E-domains (Fig. 1A). G- and E-domains are required for Gephyrin to form multimers, which are proposed to anchor inhibitory receptors at synapses [26, 27]. We generated a *gphnb* mutant in zebrafish with CRISPR/Cas9-mediated genome editing and designated this allele as *vo84*. *gphnb*^*vo84*^ represents a 5-bp deletion in exon 7 in a region encoding the G-domain, which is predicted to cause a frameshift and premature stop codon (Fig. 1A). We developed a genotyping assay that can reliably identify the homozygous mutants (Fig. 1A, B; see Methods). In the spinal cord, Gephyrin is concentrated in the neuropil regions [8, 13] (Fig. 1C, D). Immunofluorescence (IF) analysis revealed that Gephyrin signal in the neuropil of *gphnb*^*vo84/vo84*^ mutants was reduced by 89.89 ± 7.61% compared to wild-type (WT) neuropil at 5 days post-fertilization (dpf) (Fig. 1D, E). To validate our synaptic staining, we co-stained Synapsin and normalized Gephyrin signal to Synapsin signal. We found that Synapsin signals in the mutant were unchanged, and normalized Gephyrin was reduced by 92.92 ± 1.56% (Fig. 1F, G). Some *gphnb*^*vo84/vo84*^ mutants survive to adulthood, so we utilized maternal-zygotic (MZ) *gphnb*^*vo84/vo84*^ mutants to assess maternal Gephyrin contributions. We found no significant differences in the residual Gephyrin IF signal between MZ and zygotic *gphnb*^*vo84/vo84*^ mutants (Fig. 1D-G), indicating lack of maternal contribution. A previous zebrafish study using morpholinos found that knockdown of *gphna* or *gphnb* alone did not reduce Gephyrin, but knockdown of both *gphna* and *gphnb* reduced Gephyrin in the spinal cord, suggesting that *gphna* contributes to Gephyrin abundance [13]. Therefore, we sought to test if *gphna* disruption can further reduce the Gephyrin protein abundance in *gphnb*^*vo84/vo84*^. We injected two high cutting-efficiency *gphna* sgRNAs [17] with Cas9 protein into embryos, such that *gphna* would be knocked down (“crispants”). We observed neither reduction of Gephyrin IF in *gphna*^*crispant*^ larvae compared to WT nor additional reduction of Gephyrin IF in *gphnb*^*vo84/vo84*^, *gphna*^*crispant*^ larvae compared to *gphnb*^*vo84/vo84*^ mutants at 5 dpf (Fig. 1D-G), indicating a minimal or no role of *gphna* in Gephyrin abundance in the spinal cord. Together, these results suggest that the 10% remaining Gephyrin signal in these mutants likely comes from background staining. Therefore, *gphnb*^*vo84*^ is likely a null loss-of-function allele, sufficient to abolish Gephyrin abundance in the developing spinal cord of zebrafish as assessed by IF.

### *Gphnb*^*vo84/vo84*^ mutants exhibit normal gross morphology and xanthine oxidase activity

**Fig. 2:**
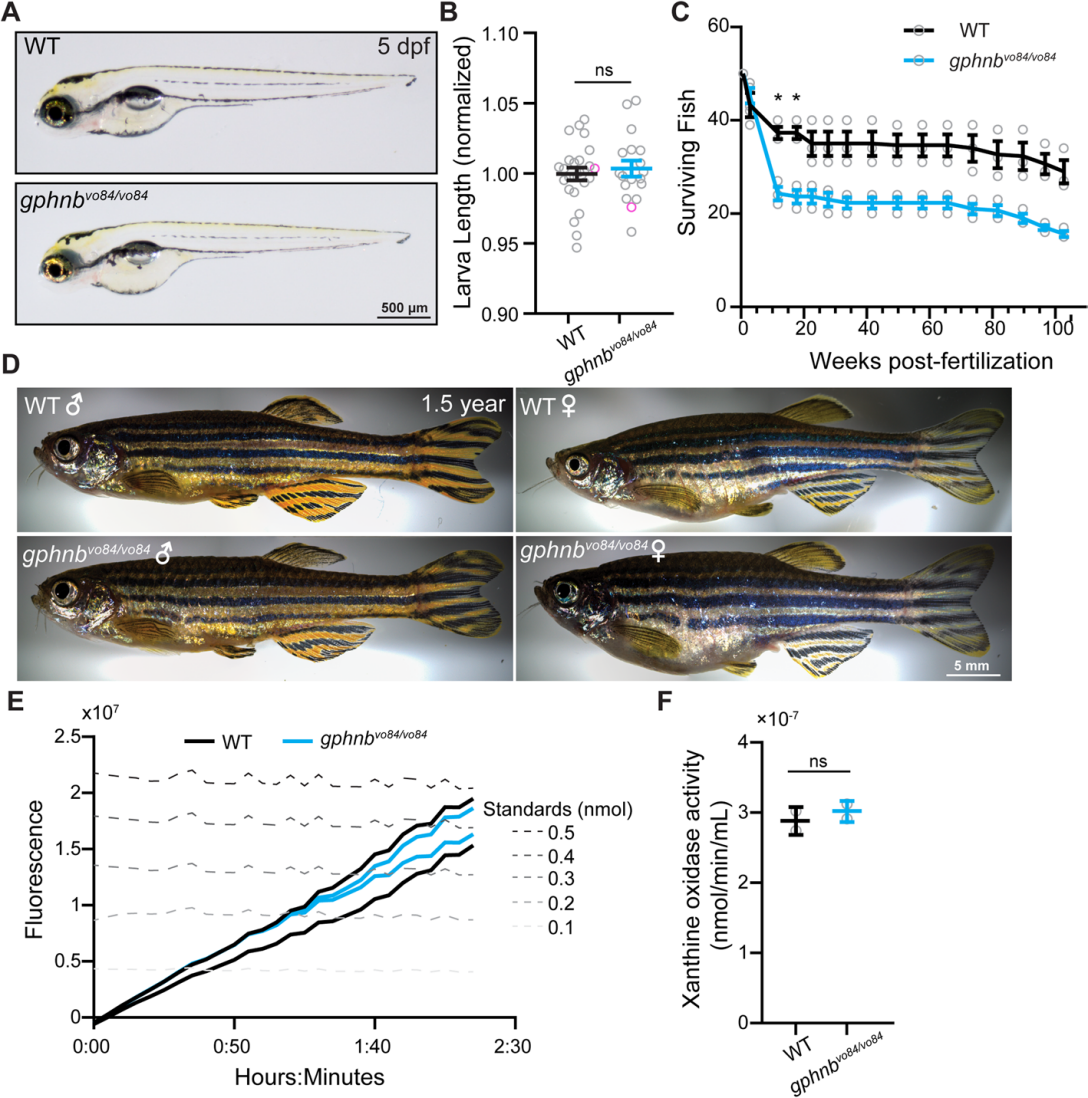
*gphnb*^*vo8/vo84*^ mutants exhibit normal gross morphology and xanthine oxidase activity. **A** Representative images of WT and *gphnb*^*vo84/vo84*^ zebrafish larvae at 5 dpf. **B** The body length of larvae at 5 dpf. n_WT_ = 13 animals, n_*gphnb*_ = 12 animals, 2 technical replicates. t[0.7040], df[23]. **C** Fish survival tracked from larvae to adults. 3 groups of 50 fish were tested per condition. F_wpf_[2.289, 9.156] = 144.2, F_genotype_[1, 4] = 20.58, F_interaction_[15, 60] = 16.14, F_tank_[4, 60] = 67.54. **D** Representative images of 1.5 years old adult zebrafish of the indicated genotypes and sexes. **E** Xanthine oxidase activity measured by fluorometric assays over time in WT and *gphnb*^*vo84/vo84*^. Each trace represents the total activity of 15 animals combined at 5 dpf. **F** Quantification of the xanthine oxidase activity at the end of recording from (E). Two sets of homogenates, each consisting of 15 animals were tested for each condition. t[0.7709], df[2]. All data are represented as mean ± SEM; ns, not significant; *, *p* < 0.05; (B,F) unpaired t-tests; (C) two-way repeat measure ANOVA with Sidak’s multiple comparison test

*Gphn*-null mice die within 1 day of birth [8], at least in part due to failures in MoCo synthesis outside the CNS. In contrast, *gphnb*^*vo84/vo84*^ developed normally at embryonic stages and exhibited normal gross morphology during larval stages (Fig. 2A, B). We observed a higher mortality of *gphnb*^*vo84/vo84*^ during late development, particularly among juveniles from 3 to 12 weeks post-fertilization (wpf) (Fig. 2C). While 74.66 ± 2.67% of WT fish survived to 12 wpf, only 48.66 ± 2.91% of *gphnb*^*vo84/vo84*^ mutants did (Fig. 2C). *gphnb*^*vo84/vo84*^ adults exhibited normal morphology (Fig. 2D) and no increased mortality (Fig. 2C). This preservation of normal development could be attributed to the presence of *gphna*, which can maintain MoCo synthesis throughout the organism. To test this, we measured the activity of a major MoCo-dependent enzyme, xanthine oxidase, previously shown to exhibit significantly reduced activity in *Gphn-*null mice [8]. We found no difference in enzyme activity between WT and *gphnb*^*vo84/vo84*^ larvae (Fig. 2E, F). Together, despite the absence of Gephyrin the spinal cord, *gphnb*^*vo84/vo84*^ mutants exhibit normal gross morphology and are capable of synthesizing MoCo.

### *gphnb*^*vo84/vo84*^ mutants exhibit swimming deficits against currents

*Gphnb*^*vo84/vo84*^ mutants offer an opportunity to study the role of Gephyrin at synapses independent of its role in MoCo synthesis. We first tested if the absence of Gephyrin in the spinal cord led to any behavioral defects. Previous studies using morpholinos to disrupt both *gphna* and *gphnb* reported defective coiling behavior (side-to-side alternating muscle contractions) at 24 h post-fertilization (hpf) and a failed touch response at 48 hpf [13]. In contrast, *gphnb*^*vo84/vo84*^ larvae exhibited normal spontaneous coiling behavior at 24 hpf (n_WT_ = 28, n_*gphnb*_ = 13; Videos 1–2) and were capable of swimming away in response to head touch at 5 dpf (n_WT_ = n_*gphnb*_ = 24).

**Fig. 3:**
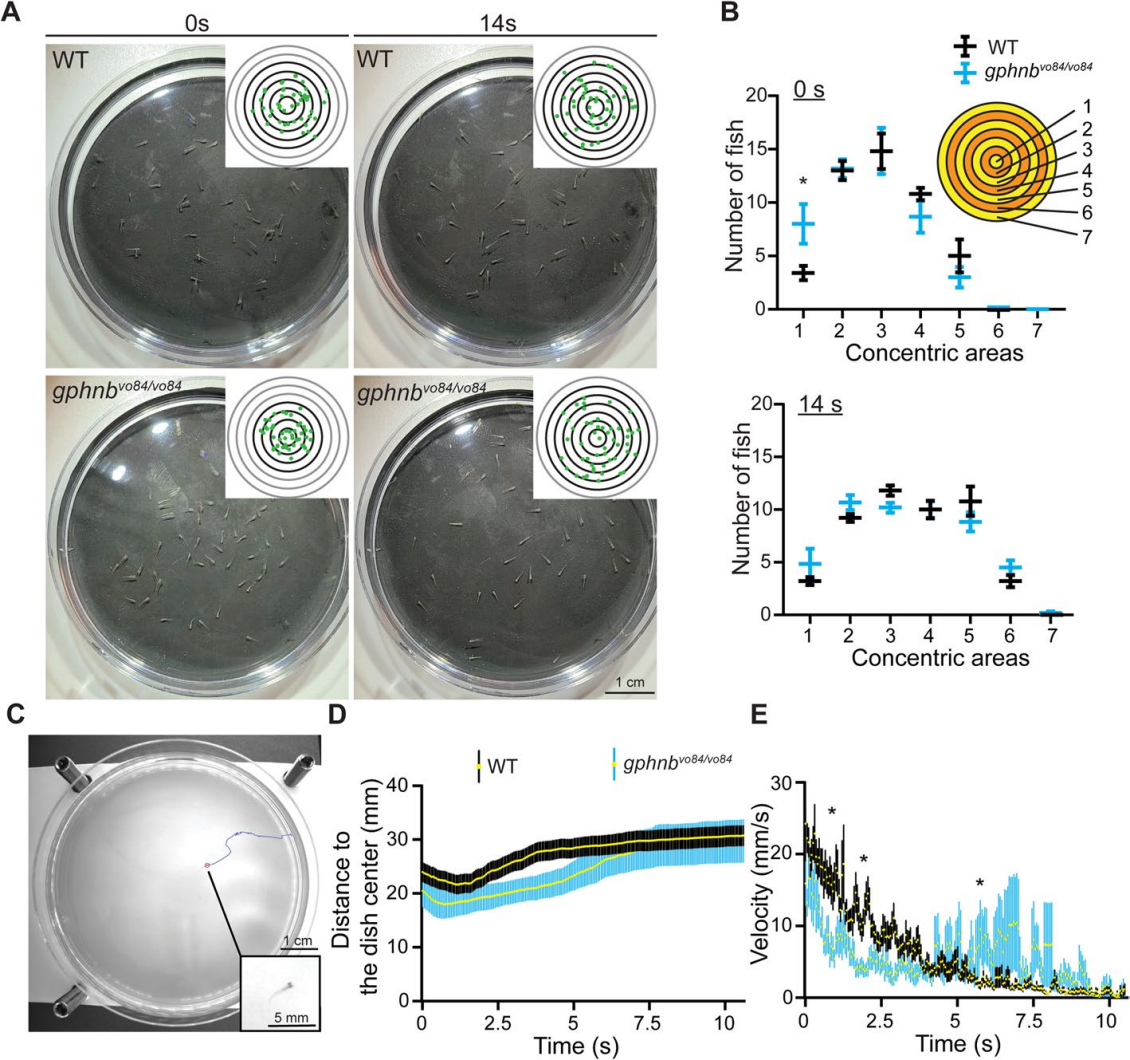
*gphnb*^*vo84/vo84*^ mutants exhibit swimming deficits against currents. **A** Representative snapshots of WT and MZ *gphnb*^*vo84/vo84*^ larvae in a forced group swimming assay at 0 and 14 s after the dish swirl. Inset shows the distribution of fish (green dots) with reference concentric regions. **B** The number of fish within concentric regions ranging from the center to the edge of a dish at 0s and 14s after the dish swirl. 5 dishes each containing 50 fish were tested per condition. F_concentric_[7, 72] = 56.91_0s_, 79.53_14s_; F_genotype_[1, 72] = 0.03419_0s_, 0.1081_14s_; F_interaction_[7, 72] = 1.670_0s_, 1.713_14s_. **C** Representative snapshots of an individual larva in a forced swimming assay with infrared light. The larva (red circle) was traced over time (blue). Inset shows the larva at higher magnification. **D** Distance of individual larvae of WT and *gphnb*^*vo84/vo84*^ from the dish center after the dish swirl. n_WT_ = 21 animals, n_*gphnb*_ = 11 animals, 1 technical replicate. **E** Swimming velocity of individual zebrafish larva after the dish swirl. n_WT_ = 21 animals, n_*gphnb*_ = 11 animals, 1 technical replicate. All data are represented as mean ± SEM; *, *p* < 0.05; (B,E) two-way ANOVA with Sidak’s multiple comparison test

In handling larvae, we noticed that *gphnb*^*vo84/vo84*^ mutants tended to cluster near the dish center after a swirl, indicating potential defects in rheotaxis (ability to swim against currents). To test this, we developed a forced group swimming assay, in which we swirled a dish containing 50 larvae, creating a current towards the center. WT larvae typically swam against the current and distributed relatively evenly in the dish (Fig. 3A, B and Video 3). However, *gphnb*^*vo84/vo84*^ larvae clustered closer to the center immediately after the swirl (Fig. 3A, B and Video 4). To further analyze this behavior, we customized a dark chamber and tracked individual zebrafish with infrared light to eliminate any influence from visible light (Fig. 3C and Videos 5). In this forced individual swimming assay, *gphnb*^*vo84/vo84*^ mutants also localized closer to the dish center after the swirl (Fig. 3D and Video 6). The swimming velocity of individual *gphnb*^*vo84/vo84*^ fish was slower than that of WT fish initially (Fig. 3E), likely contributing to their centered distribution after swirls. Interestingly, these mutants exhibited a burst of speed a few seconds later when the current subsided (Fig. 3E), suggesting that the current suppressed the swimming ability of *gphnb*^*vo84/vo84*^ animals. Together, these data suggest that synaptic Gephyrin in the spinal cord is dispensable for coiling behavior and touch response but plays an important role in rheotaxis.

### Cell densities and myelin are unchanged in the spinal cord of *gphnb*^*vo84/vo84*^ mutants

**Fig. 4:**
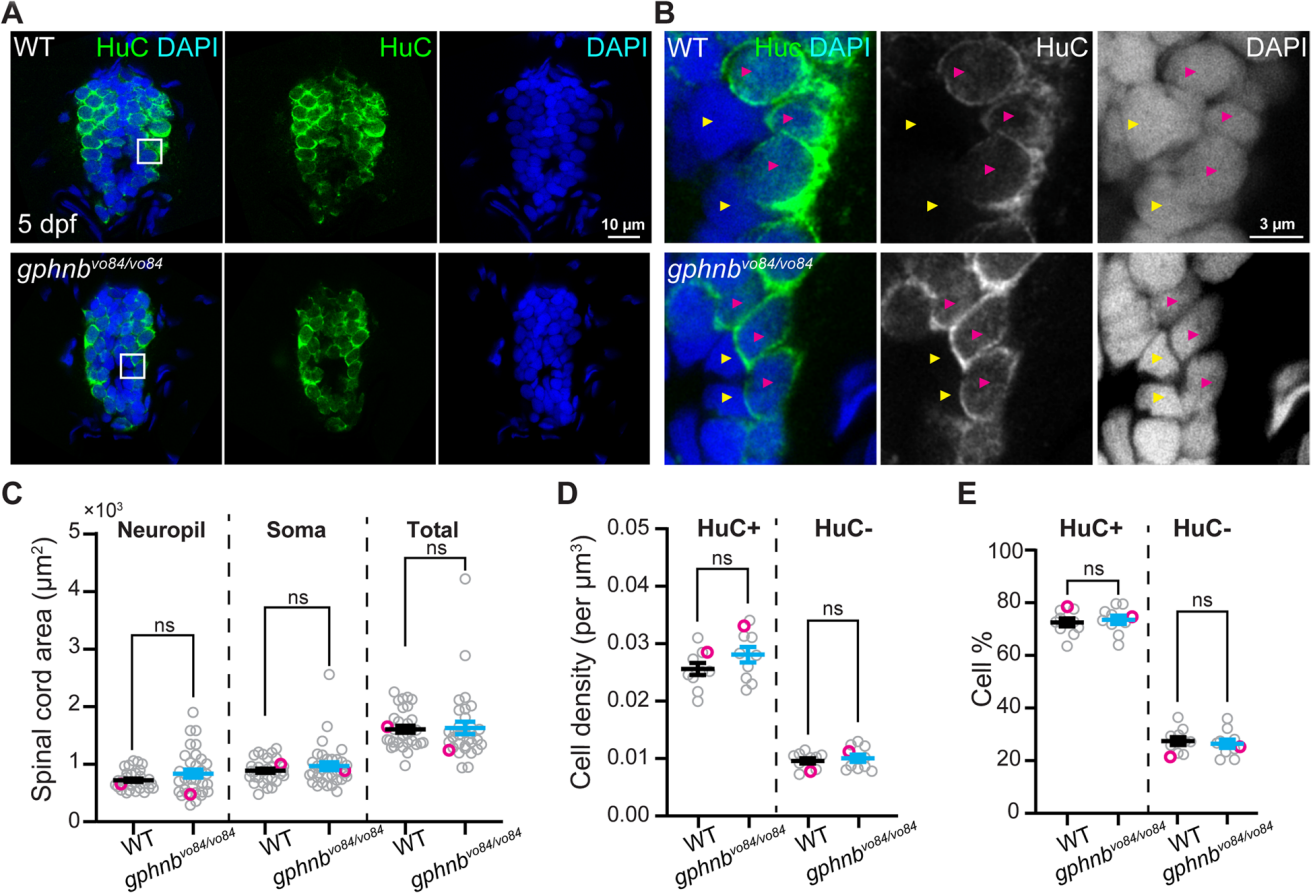
Cell densities are unchanged in *gphnb*^*vo84/vo84*^ mutant spinal cord. **A** Representative single-plane images of transverse larval spinal cord sections of WT and *gphnb*^*vo84/vo8*^ with HuC (green) and DAPI (blue) staining at 5 dpf. **B** Zoom-in image from (A) showing HuC+ (magenta arrowheads) and HuC- (yellow arrowheads) cells. **C**-**E** Quantifications of (**C**) transverse spinal cord area, (**D**) cell density, and (E) cell percentage from (**A**, **B**). **C** n_WT_ = 22 fish, 31 sections; n_*gphnb*_ = 20 fish, 32 sections; 6 technical replicates; t_neuropil_[1.483], t_soma_[1.021], t_total_[0.1881], df[61]. (D,E) n = 7 fish, 10 sections per condition; 1 technical replicate; t_HuC+density_[1.619], t_HuC-density_[0.5591], t_%_[0.4604], df[18]. All data are represented as mean ± SEM; ns, not significant; (C-E) unpaired t-tests

**Fig. 5:**
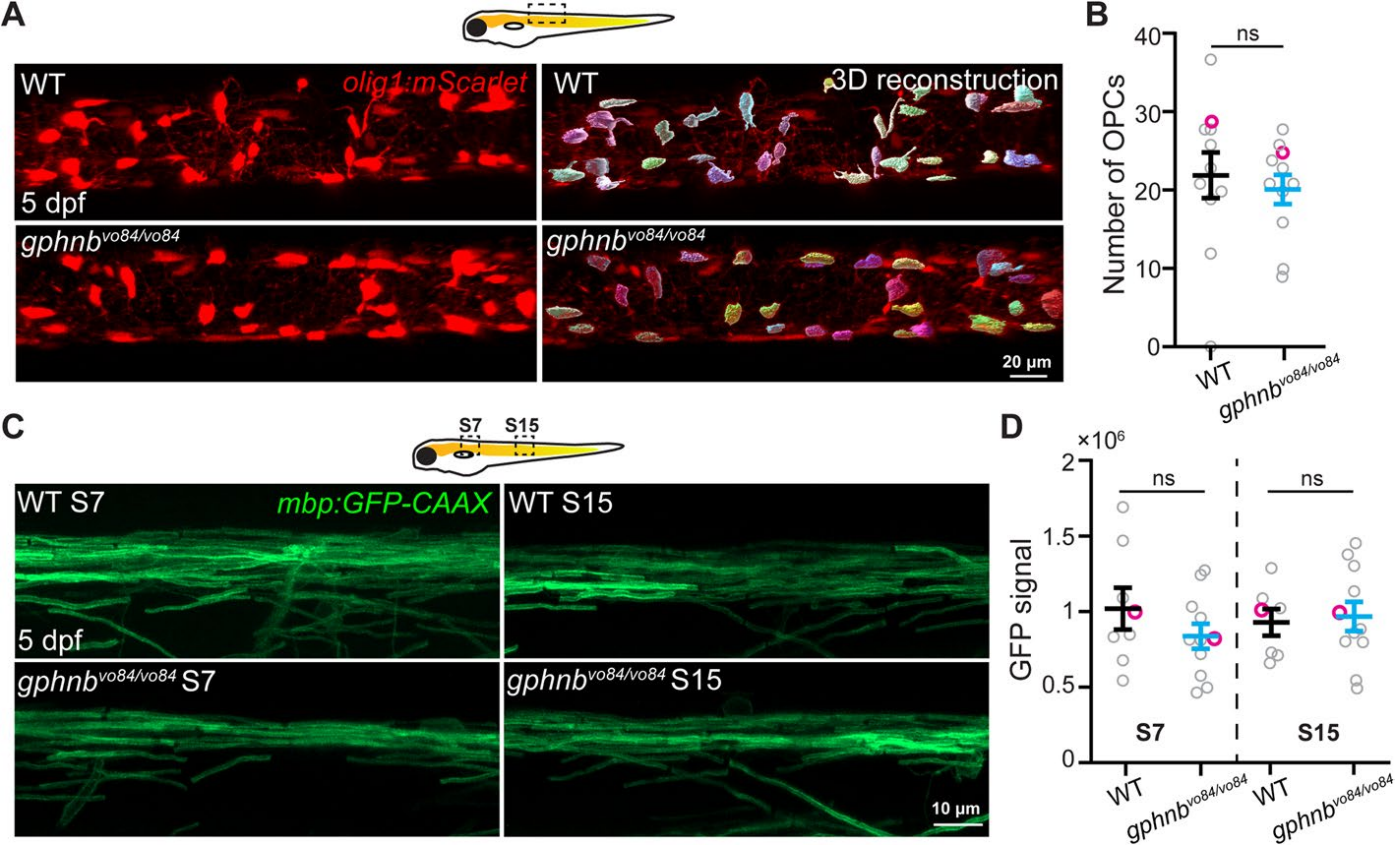
Oligodendrocyte precursors and myelin are unchanged in *gphnb*^*vo84/vo84*^ mutant spinal cord. **A** Representative in vivo images from *Tg(olig1:mScarlet)* and 3D reconstruction showing individual OPCs in the spinal cord of WT and *gphnb*^*vo84/vo84*^ at 5 dpf. **B** OPC number in 300-µm-length spinal cord from (**A**). n_WT_ = 11 animals, n_*gphnb*_ = 11 animals, 1 technical replicate, t[0.5192] df[20],. **C** Representative in vivo images of myelin in the dorsal spinal cord from *Tg(mbp:GFP-CAAX)* at body segment 7 (S7) and 15 (S15) of WT and *gphnb*^*vo84/vo84*^ at 5 dpf. **D** GFP signal intensity from (**C**). n_WT_ = 8 animals, n_*gphnb*_ = 11 animals, 1 technical replicate, F[3,, 33] = 0.6057. All data are represented as mean ± SEM; ns, not significant; (**B**) unpaired t-tests; (**D**) one-way ANOVA with Tukey’s post-hoc test

We wondered if the rheotaxis defects observed in *gphnb*^*vo48/vo84*^ mutants could be attributed to cellular changes in the spinal cord. Examination of spinal cord sections from *gphnb*^*vo84/vo84*^ animals revealed similar area of neuropil and soma regions compared to WT (Fig. 4A-C). Subsequently, we assessed cell density in the spinal cord using the nuclear marker DAPI and the neuronal marker HuC (Fig. 4A, B). Our analyses indicated that overall cell densities and percentage of neurons (HuC+) and non-neurons (HuC-) were comparable between WT and *gphnb*^*vo84/vo84*^ animals (Fig. 4D, E). Given the role of myelin in animal movement [28],the recent association of Gephyrin with myelination [17], and white matter lesions in MoCo deficiency [3, 4], we next examined oligodendrocyte development and myelination. Our findings revealed similar numbers of oligodendrocyte precursor cells (OPCs) in WT and *gphnb*^*vo84/vo84*^ spinal cords (Fig. 5A, B). Myelination in *gphnb*^*vo84/vo84*^, evaluated using the transgenic reporter *Tg(mbp:GFP-CAAX)*, also appeared normal (Fig. 5C, D). These results suggest that overall cell density and myelination are not affected in the spinal cord of *gphnb*^*vo84/vo84*^mutants.

### GlyRs are reduced, but remain proximal to synaptic regions in the spinal cord of *gphnb*^*vo84/vo84*^ animals

**Fig. 6:**
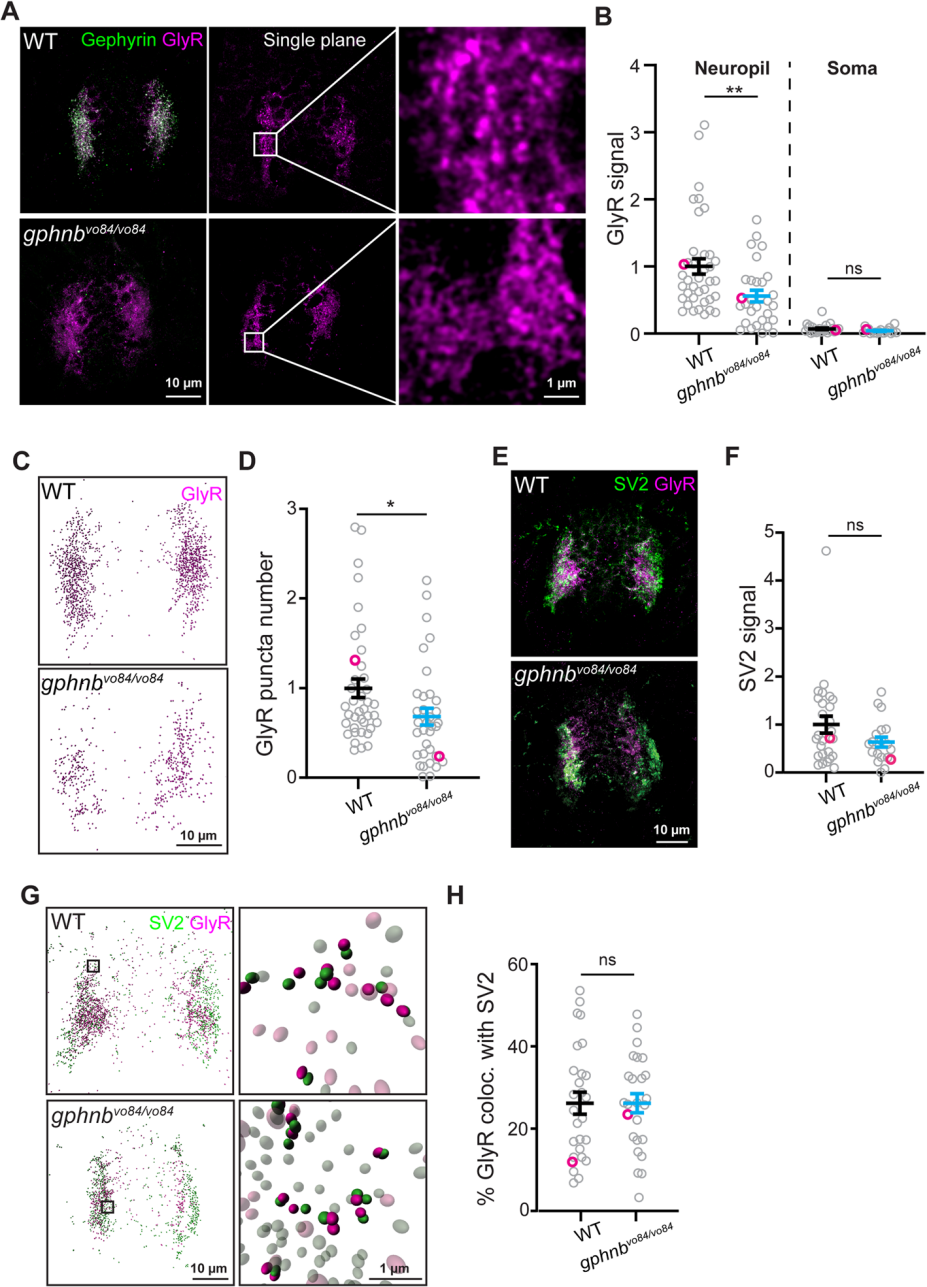
GlyRs are reduced, but remain proximal to synaptic regions in the spinal cord of *gphnb*^*vo84/vo84*^ animals. **A** Representative transverse images of Gephyrin and GlyR staining from WT and *gphnb*^*vo84/vo84*^ at 5 dpf. From left, maximum z-projection, single plane, and zoom-in images. **B** GlyR signal normalized to WT from (A). n_WT_ = 14 fish, 38 sections, n_*gphnb*_ = 15 fish, 29 sections, 3 technical replicates, t_neuropil_[2.931] df[65], t_soma_[0.2904] df[19]. **C** 3D reconstruction of GlyR from (A) with Imaris. **D** GlyR puncta number in 3D reconstruction from (C). n_WT_ = 14 fish, 39 sections, n_*gphnb*_ = 15 fish, 35 sections, 3 technical replicates, t[2.239], df[72]. **E** Representative transverse images of SV2 and GlyR staining from WT and *gphnb*^*vo84/vo84*^ at 5 dpf (maximum z-projection). **F** SV2 signal normalized to WT from (**E**). n_WT_ = 7 fish, 19 sections, n_*gphnb*_ = 8 fish, 27 sections, 2 technical replicates, t[1.602], df[44]. **G** 3D reconstruction of SV2 and GlyR from (E) with Imaris with zoom-in images on the right. In the zoom-in images, colocalizing puncta are in solid color and non-colocalizing puncta are transparent. **H** The percentage of GlyR that colocalize with SV2 from (G). n_WT_ = 7 fish, 27 sections, n_*gphnb*_ = 8 fish, 25 sections, 2 technical replicates, t[0.004848], df[50]. All data are represented as mean ± SEM; ns, not significant; *, *p* < 0.05; **, *p* < 0.01; (B,D,F,H) unpaired t-tests

In *Gphn*-null mice, GlyRs are diffuse, absent from synapses, and do not colocalize with presynaptic markers [8]. To test if *gphnb*^*vo84/vo84*^ mutants possessed similar defects, we examined the abundance and localization of GlyRs in the spinal cord at 5 dpf with antibody staining. Interestingly, GlyRs still localized to the neuropil regions of *gphnb*^*vo84/vo84*^ spinal cord (Fig. 6A). We did observe a 44.15 ± 15.06% reduction in GlyR levels in the neuropil of *gphnb*^*vo84/vo84*^ animals compared to WT (Fig. 6A, B). Minimal GlyR signal was observed in the soma region, showing no significant differences between WT and *gphnb*^*vo84/vo84*^ zebrafish (Fig. 6A, B). We further identified each individual GlyR puncta using Imaris and observed a 31.70 ± 14.15% reduction in puncta number in the spinal cord of *gphnb*^*vo84/vo84*^ mutants compared to WT (Fig. 6C, D). To further investigate if the GlyRs localize to synapses, we examined GlyRs’ colocalization with presynaptic marker SV2. Similar to previous *Gphn*-null mice [8], SV2 abundance was not affected in the spinal cord (Fig. 6E, F). Surprisingly, we found similar levels of colocalization of SV2 and GlyRs in both WT and *gphnb*^*vo84/vo84*^ animals (0.5 μm distance threshold; WT, 26.18 ± 2.68%; *gphnb*^*vo84/vo84*^, 26.20 ± 2.32%) (Fig. 6G, H), indicating that the remaining GlyRs can localize to synaptic regions in the absence of Gephyrin. Together, these results suggest that while GlyRs are reduced in the neuropil of *gphnb*^*vo84/vo84*^, they remain in close proximity to synaptic regions.

## Discussion

Postsynaptic organization plays a pivotal role in synaptic function and plasticity at inhibitory synapses [1]. Gephyrin acts as the postsynaptic scaffold, interacting with a myriad of proteins to enable inhibitory synaptic transmission. Among these interactions, Gephyrin’s association with GlyRs is best known, as exemplified in foundational work co-purifying Gephyrin and GlyRs from spinal cords using strychnine affinity columns [29, 30]. Currently, Gephyrin is thought to directly regulate GlyR clustering at postsynaptic sites, as best shown by the absence of GlyRs at synapses in *Gphn*-null mice [8]. However, assessing Gephyrin’s specific synaptic function has been challenging due to its dual role as a critical enzyme for MoCo synthesis. Indeed, the *Gphn*-null mice die perinatally, complicating the interpretation of the synaptic phenotypes.

In zebrafish, Gephyrin is duplicated and expressed in various tissues. Here, we report a new mutant allele, *gphnb*^*vo84*^, that led to nearly complete absence of Gephyrin protein in the spinal cord. With maternal depletion and additional *gphna* disruption, we did not observe further Gephyrin reduction, establishing *gphnb* as the source for Gephyrin in the spinal cord. Interestingly, these animals appear grossly normal at larval and adult stages and exhibit normal activity of xanthine oxidase and neuronal densities. Unlike *Gphn*-null mice or zebrafish with morpholinos targeting both *gphna* and *gphnb* [8, 13], *gphnb*^*vo84/vo84*^ mutants show overall normal movement and response to touch. These data suggest that the lack of synaptic Gephyrin in the spinal cord does not cause perinatal lethality or severely impaired movement. Instead, the reduced MoCo enzymatic activity of Gephyrin in other cell types and tissues possibly mediates these defects, consistent with the early lethality and neuronal death in *Mocs1* and *Mocs2* mice [5–7]. Transcriptomic data from mice reveal that *Gphn* is also expressed in OPCs, oligodendrocytes, and astrocytes [31, 32]. These CNS glia have recently been linked to movement disorders, such as Parkinson’s disease [33, 34]. It would be intriguing to explore how Gephyrin in glia or other cell types contribute to movement and response to touch in future studies.

We observed rheotaxis defects in *gphnb*^*vo84/vo84*^ larvae. Rheotaxis relies on lateral line neurons to detect currents [35–37]. These sensory inputs are then processed in the hindbrain, involving inhibitory neurons and resulting in distinct motor outputs between the left and right sides of the body [38]. Our observations of reduced GlyRs in *gphnb*^*vo84/vo84*^ larvae likely impaired the function of inhibitory transmission within this circuit, disrupting the correct sequence of muscle contraction and relaxation between the two sides of the body. Alternatively, it is possible that a “suppressive” signal is produced in the brain in response to currents in the mutants and interferes with proper rheotaxis. We suspect that such defects in rheotaxis may contribute to the increased mortality in late development of the mutants. In surviving adults, we did not observe obvious swimming defects in *gphnb*^*vo84/vo84*^ mutants. One possibility is that the increased mortality in late development of the mutants might lead to selective survival of healthier animals, potentially masking behavior defects in adults. Therefore, future directions rescuing the late development mortality (*e.g.*, reducing water flow) might provide more insight into the role of Gephyrin in adult behaviors. In addition, it would be interesting to test if other types of directional movement are also affected in these mutants.

Mutations in *GPHN* and *GLRA1* (Glycine receptor alpha 1) have been linked to hyperekplexia in humans, a disorder that manifests from childhood and is characterized by exaggerated startle responses, stiffness, and an inability to move [39, 40]. Interestingly, in our swimming assay, we observed that *gphnb*^*vo84*^ larvae initially exhibited impairment in response to currents (within 2.5 s), followed by a burst of activity thereafter (5–10 s). We speculate that such distinct phases of activity also occur in hyperekplexia. It is worth noting that reduced GlyRs can affect not only neurons but also glia [41, 42]; yet the role of glia in hyperekplexia remains unclear. To better characterize this disease and dissect its underlying mechanisms, zebrafish could provide the necessary tools to combine in vivo imaging and behavior assays to assess the underlying circuits from early developmental stages.

Surprisingly, in *gphnb*^*vo84/vo84*^ animals, the remaining GlyRs still localize to the synaptic regions and exhibit a similar colocalization ratio with SV2. We noted that only a portion of GlyRs colocalize with SV2 in WT, indicating SV2 may not label all inhibitory presynaptic terminals. Nonetheless, these results challenge the previous notion that the depletion of Gephyrin at synapses would inevitably result in diffuse and non-synaptic GlyR localization. While electron microscopy would offer the resolution needed to precisely define the synaptic location of GlyRs, our results suggest that GlyRs are transported to axons and are capable of forming clusters near synapses even in the absence of Gephyrin. Overall, this viable *gphnb*^*vo84*^ allele provides an excellent tool for studying the synaptic function of Gephyrin without the confounding factors of major defects in animal health.

## Conclusions

We identified a viable zebrafish *gphnb* mutant that shows nearly no defects that were previously associated with MoCo deficiency. Instead, this mutant exhibits impaired rheotaxis. Moreover, GlyRs still cluster to some degree in the synaptic regions of *gphnb* mutant, contrasting the diffuse GlyRs observed when both *gphna* and *gphnb* were disrupted in previous studies [13]. These observations underscore the distinct roles of Gephyrin in synaptic function and MoCo synthesis.

### Supplementary Information

Additional file 1: Video 1. Spontaneous coiling behavior of WT larva at 1 dpf.

Additional file 2: Video 2. Spontaneous coiling behavior of *gphnb*^*vo84/vo84*^ larva at 1 dpf.

Additional file 3: Video 3. WT larvae in forced group swimming assay at 5 dpf.

Additional file 4: Video 4. MZ *gphnb*^*vo84/vo84*^larvae in forced group swimming assay at 5 dpf.

Additional file 5: Video 5. WT larva in individual forced swimming assay at 5 dpf with path tracked.

Additional file 6: Video 6. *gphnb*^*vo84/vo84*^larva in individual forced swimming assay at 5 dpf with path tracked.

## Data Availability

All data used during the current study are available from the corresponding author on reasonable request.

## Abbreviations

CNS: Central nervous system
MoCo: Molybdenum cofactor
GlyR: Glycine receptor
*gphnb*: *gephyrinb*
*gphna*: *gephyrina*
WT: Wild-type/wildtype
hpf: Hours post-fertilization
dpf: Days post-fertilization
wpf: Weeks post-fertilization
MZ: Maternal-zygotic
OPC: Oligodendrocyte precursor cell
*Mbp*: *Myelin* *basic* *protein*
